# Thermal stress and thermal deformation of a nozzle flowmeter with different weld structures

**DOI:** 10.1371/journal.pone.0324780

**Published:** 2025-06-16

**Authors:** Hai-Bing Jiang, Ze-Zhou Yang, Yu-Liang Zhang, Xiao-Wei Xu, Yan-Juan Zhao

**Affiliations:** 1 College of Mechanical Engineering, Quzhou University, Quzhou, China; 2 College of Mechanical Engineering, Zhejiang University of Technology, Hangzhou, China; 3 College of Information Engineering, Quzhou College of Technology, Quzhou, China; G H Raisoni College of Engineering and Management, Pune, INDIA

## Abstract

Nozzle flow meters are widely used in the field of measuring high temperature media. In order to investigate the effect of nozzle flowmeter weld structure on thermal stress and thermal deformation, 10 different weld structures were designed in terms of the distance between the weld metal and the eight-groove nozzle, the width of the bottom of the weld metal, the taper angle of the weld metal, and the opening of the taper angle of the weld metal. The thermal stress and thermal deformation characteristics of the solid domain of the nozzle flowmeter are also calculated numerically for a high temperature case. The conclusion indicates that with the increase of the conical Angle of the weld metal, the thermal stress at the inlet and outlet of the nozzle flowmeter gradually decreases, and the thermal deformation changes less. The distance between the weld metal of the nozzle flowmeter and the eight-slot nozzle, the width below the weld metal, and the variation of the cone angle opening of the weld metal all have a relatively small impact on the thermal stress and deformation of the overall solid domain of the nozzle flowmeter.

## 1. Introduction

Nozzle flowmeter is a differential pressure generating device used to measure the flow rate of various fluids in pipelines, and is widely used in production practices in various industrial sectors such as petroleum, power, and light industry. When the liquid flows through the nozzle of the flowmeter, the static pressure difference is generated in the upstream and downstream of the nozzle, thereby achieving the function of measuring fluid flow through the nozzle flow meter. So far, many scholars have studied the characteristics of various flowmeters under different working conditions and different media. Many scholars have also developed updated and more comprehensive measurement methods for flow meter characteristics. In many special fields, scholars have designed new flow meters that are more suitable for work needs when existing flow meters are no longer able to meet their usage needs.

Some scholars have made research and innovation in the field of nozzle flowmeter in recent years. Li et al. numerically calculated the transient thermal characteristics of the various components of the nozzle flowmeter, and found that there were high thermal stress zones and high thermal deformation zones near the upstream and downstream pressure tapping points. While the high thermal stress zones also existed near the inlet and outlet of the flowmeter, and the high thermal deformation zones also existed near the eight-groove nozzle of the flowmeter [1]. Zhang et al. made a study on the thermal effect of the nozzle flowmeter at five different inner wall temperatures. They concluded that as the inner wall temperature increases, the thermal stress increases, and in the upstream and downstream pressure inlet and flowmeter inlet and outlet areas produce obvious stress concentration phenomenon by numerical simulation [2]. Oh et al. have made compare and analysis of several typical gas flowmeters, and developed a portable multi-nozzle flowmeter with outlet pressure control, which is easier to operate and more accurate than other typical flowmeters [3]. Zhang et al. used numerical calculation to study the thermal characteristics of small nozzle flowmeters when conveying medium with different temperatures. The study found that when conveying high temperature medium, there was obvious temperature stratification near the inner wall of the flowmeter, and the heat flow field at the inlet and outlet of the flowmeter increased significantly with increasing of temperature [4].

Other scholars have revealed the various properties of ultrasonic flowmeters for us. Zheng et al. found that the measurement error of gas ultrasonic flow meters exhibits significant nonlinearity with the increase of flow rate. To address this, a correction model was proposed to improve the nonlinearity of the measurement error [5]. Hamouda et al. proposed a new water flow measurement method suitable for ultrasonic flow meters, which can achieve smaller minimum detectable flow rates compared to previous methods [6]. Chen et al. designed a new type of filtering ultrasonic gas flow meter, which adopts a new data filtering algorithm, which improves the measurement accuracy compared to typical ultrasonic gas flow meters, and also has characteristics such as good stability and low power consumption [7]. Franklin et al. developed a pulse ultrasonic flowmeter for measuring blood flow in the main blood vessels of anesthetized animals. The flowmeter functions by outputting voltage to represent volumetric flow, and can respond to step changes in flow within 10 milliseconds [8]. Mandard et al. studied a method for developing high-precision ultrasonic transfer time flow meters specifically for liquid hydrocarbons and analyzed various methods for achieving high precision. The results of the final simulations and experiments show that this method can characterise the flow under disturbed and undisturbed flow conditions [9]. Willigen et al. proposed an algorithm to minimize the zero flow error of real-time ultrasonic flow meters by combining the advantages of cross correlation method and zero crossing detection method. This algorithm can be adjusted according to the constantly changing zero flow error, and can greatly reduce the zero flow error without increasing circuit complexity [10]. Peng et al. proposed a data fusion based calculation method for estimating flow velocity in multipath ultrasonic flow meters. This method determines the weight based on the variance of the data measured from each channel, and can obtain the fusion flow velocity with the minimum mean square error [11]. Buess et al. designed a new type of ultrasonic flowmeter, which has the characteristics of fast speed, high accuracy, low noise, and wide flow range. The linear frequency can reach 70 Hz [12]. Chen et al. optimized and improved an ultrasonic flow meter for liquid flow measurement in the semiconductor manufacturing field. Through testing, the accuracy of this improved flow meter reached level 1.5, which better meets the work requirements [13].

Some scholars have explored the field of electromagnetic flow meters and have successfully reached many important conclusions. Kolin developed a method for tracking rapid flow changes based on the principle of electromagnetic induction and verified this method through experiments. The induced voltage of this electromagnetic flowmeter is proportional to the liquid flow rate, and it has the advantage of a linear relationship between deflection and flow rate [14]. Jiang proposed a new method for calculating the weight function of an electromagnetic flowmeter for gas-liquid two-phase flow. The rationality of this method was verified through experiments, and the results showed that the proposed method can accurately describe the impact of the gas contained in the measured fluid on the output signal of the sensor [15]. Jin et al. combined with a plug-in conductance sensor array (PICSA) to study the characteristics of an electromagnetic flowmeter for the measurement of low-speed oil-water two-phase flow. The results show that the correlation between electromagnetic field output and water retention is quite sensitive to changes in flow patterns. Meanwhile, their team used this method to predict the surface velocity of the oil layer and obtained high-precision data results [16]. Ge et al. analyzed and studied the interference and noise issues of electromagnetic flow meters, and proposed a new design scheme for electromagnetic flow meters based on the principle of differential correlation detection. The experimental conclusion shows that the electromagnetic flowmeter of this design scheme can still meet the working requirements for detection in strong noise, low flow, and mud flow environments [17]. Cheng et al. designed a portable intelligent electromagnetic flowmeter for coal mines, which uses high-precision chips and has the advantages of high measurement accuracy and low power consumption. The experimental results show that the flowmeter has good accuracy and can meet the needs of long-term operation in coal mines [18].

There are also scholars who have studied many aspects of various other flowmeters. Song et al. developed an image-based optical fluid flowmeter, which is a polymer based sensing chip that can be mass-produced at an extremely low cost. It can also be integrated into a lab-on-a-chip system for on-site measurement of air pressure and flow rate [19]. Tong et al. conducted a study on the safety performance of industrial throttle valve flow meters during welding. residual stress at dissimilar steel welds was tested and analyzed through the self-developed residual stress distribution calculation software. The results showed that the residual stress at the weld of dissimilar steel was large, and the mechanical property test was generally unqualified [20]. Shang et al. developed a C++ based simulation software for ultrasonic inspection of flowmeter welds, which can efficiently screen out all probes with appropriate K values. Through experiments, the thickness of the weld seam was scanned on the artificial defect test block of the flow meter to ensure the reliability of the software [21]. Phan et al. developed a gas flow meter that has the characteristics of low pressure loss and self power supply. They also studied the effects of membrane material, geometric shape, and flow humidity on the flow meter [22]. Weathered et al. designed a permanent magnet flowmeter and validated it with a finite element model. This flowmeter is suitable for immersion in a sodium fast reactor environment to determine the sodium flow rate in the thermal hydraulic experimental testing loop [23]. Pankanin provides an in-depth study of Karman vortex flowmeters, giving a comprehensive overview of the physical characteristics, the research process and the application environment of such flowmeters, as well as an overview of the methodology used to study various phenomena in vortex flowmeters [24]. Hosseini et al. improved the gas-liquid two-phase flow meter used in the petroleum and petrochemical industry, calculated the efficiency of multiphase flow meters using MCNP, and conducted in-depth analysis using wavelet transform and feature extraction methods to improve the efficiency of flow meters [25]. Thess et al. proposed the Lorentz force flowmeter theory, which links the measured force with the unknown flow rate, and proposed a general kinematic theory applicable to any magnetic material or current distribution and any velocity distribution, providing a framework for predicting the sensitivity of Lorentz force flowmeters [26]. Li et al. proposed a novel method for wet gas flow measurement that combines a vortex flowmeter with an interfering wave frequency, which has low medium conductivity requirements and can be applied to a wider variety of media [27]. Compared with the internal flow inside fluidmachinery, the thermal characteristics is more complicated [28–34].

From the published literature, most scholars have conducted systematic research on various aspects of flow meters. However, it was found that the influence of different weld structures on the thermal stress and deformation of nozzle flow meters under high temperature conditions has not yet been explored. This article uses numerical simulation methods to conduct numerical calculations on the influence of different weld structures on the heat flow field of a certain model of nozzle flowmeter, aiming to obtain the distribution characteristics of thermal stress and thermal deformation of nozzle flowmeter under different weld structures.

## 2. Calculation model and method

### 2.1 Flowmeter model

**Fig 1:**
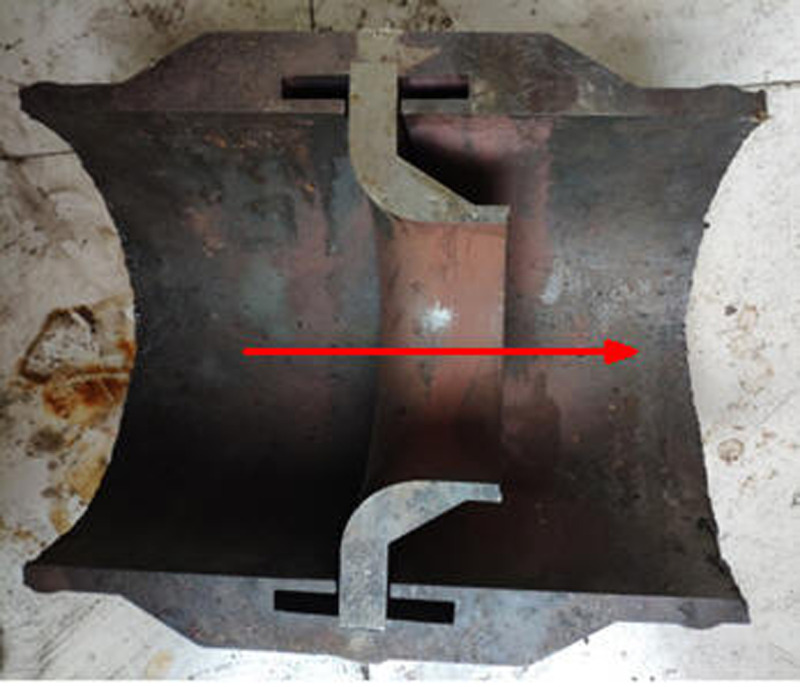
Overall structure [35]. (a)real products (b)basic structure.

The nozzle flowmeter is mainly composed of a front measuring tube, a rear measuring tube, a nozzle, and weld metal. The structure of the flowmeter is shown in Fig 1, and detailed geometric parameters can be found in the reference [35]. The measuring tube is divided into a front measuring tube and a rear measuring tube, which are connected by an eight slot nozzle and weld metal. The material of the front and rear measuring tubes is 12Cr1MoV, the material of the eight slot nozzle is structural steel, and the material of the weld metal is tin. The specific attributes are shown in Table 1.

**Table 1:** Material properties [36].

Materials	Density (kg/m^3^)	Isotropic Thermal Conductivity (W/(m∙K))	Specific Heat (J/(kg∙K))
12Cr1MoV	7860	60.5	434
Structural Steel	7850	60.5	434
Tin	7304	64	226.5

### 2.2 Numerical simulation method

**Fig 2:**
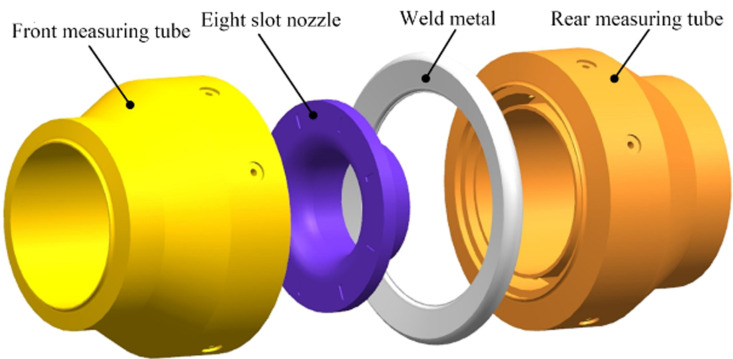
Schematic diagram of solid structure and flow field calculation domain. (a) Flow meter structure (b) Solid field structural domain grid.

3D modeling was carried out for each component of the nozzle flowmeter, as shown in Fig 2 (a); This article uses ICEM software to perform grid partitioning, using unstructured tetrahedral meshes with good adaptability, and completes grid independence verification [35]; The original structure of the flow meter includes 63875, 63872, 1424041, and 38623 grids for the front measuring tube, rear measuring tube, eight slot nozzle, and weld metal watershed, respectively. The total number of grids is 1590411, and the grid division results are shown in Fig 2 (b). The numerical calculation of the structural domain of the nozzle flowmeter was completed using the solid field finite element analysis software ANSYS Workbench.

### 2.3 Calculation scheme

**Fig 3:**
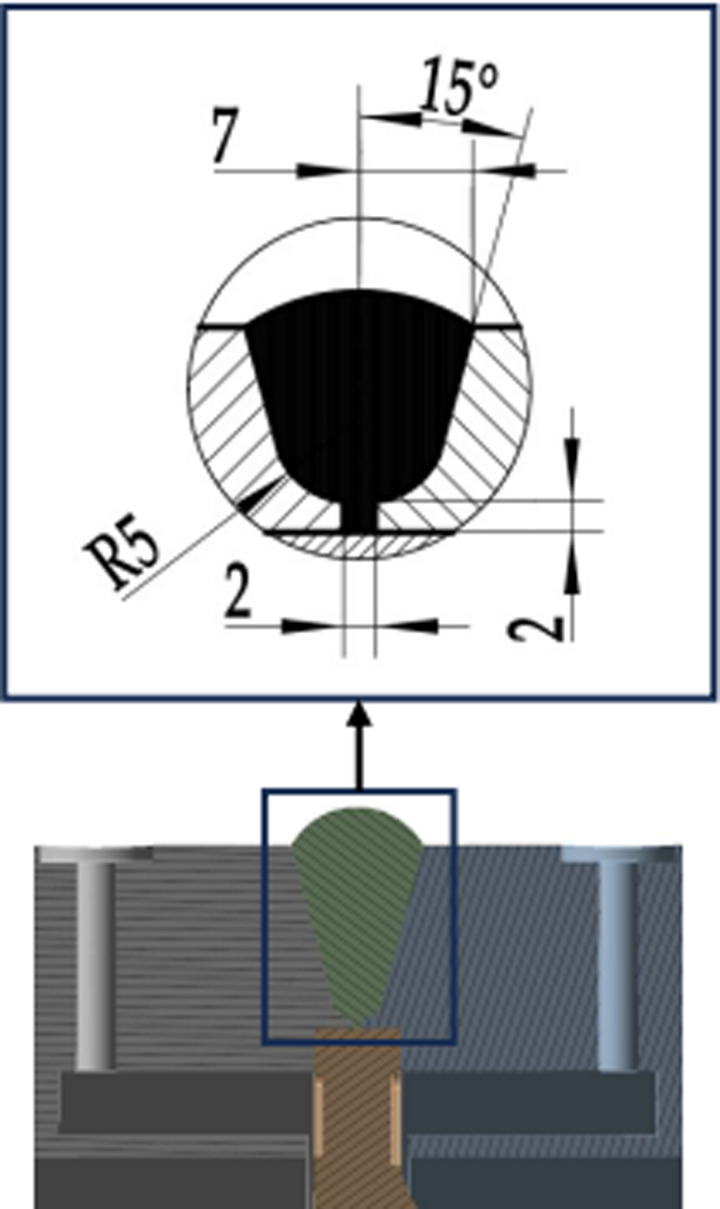
Section of Type I weld structure.

**Fig 4:**
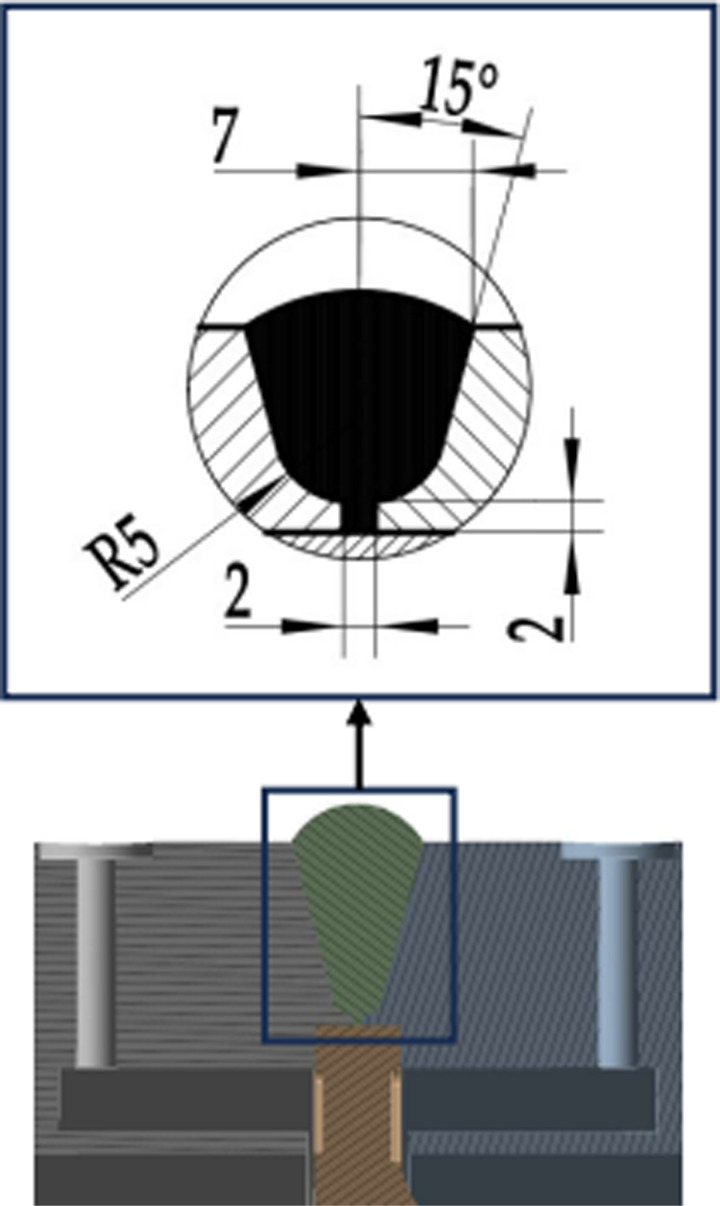
Section of Type Ⅱ weld structure.

**Fig 5:**
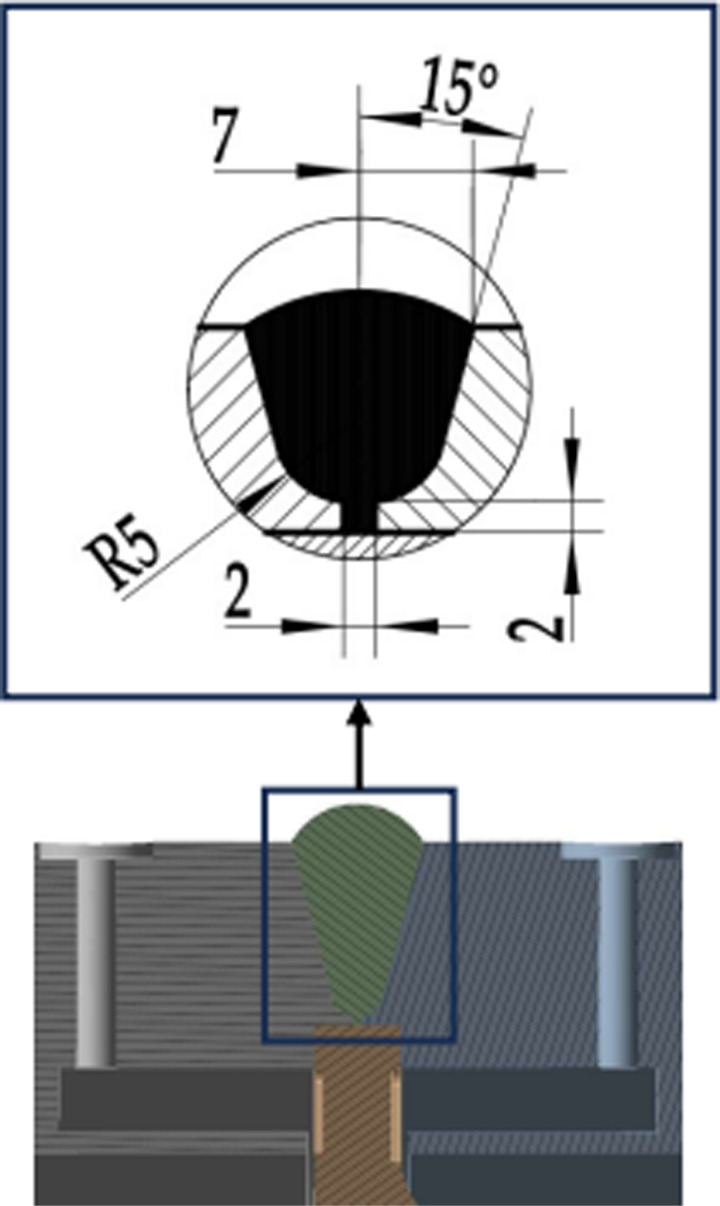
Section of Type Ⅲ weld structure.

**Fig 6:**
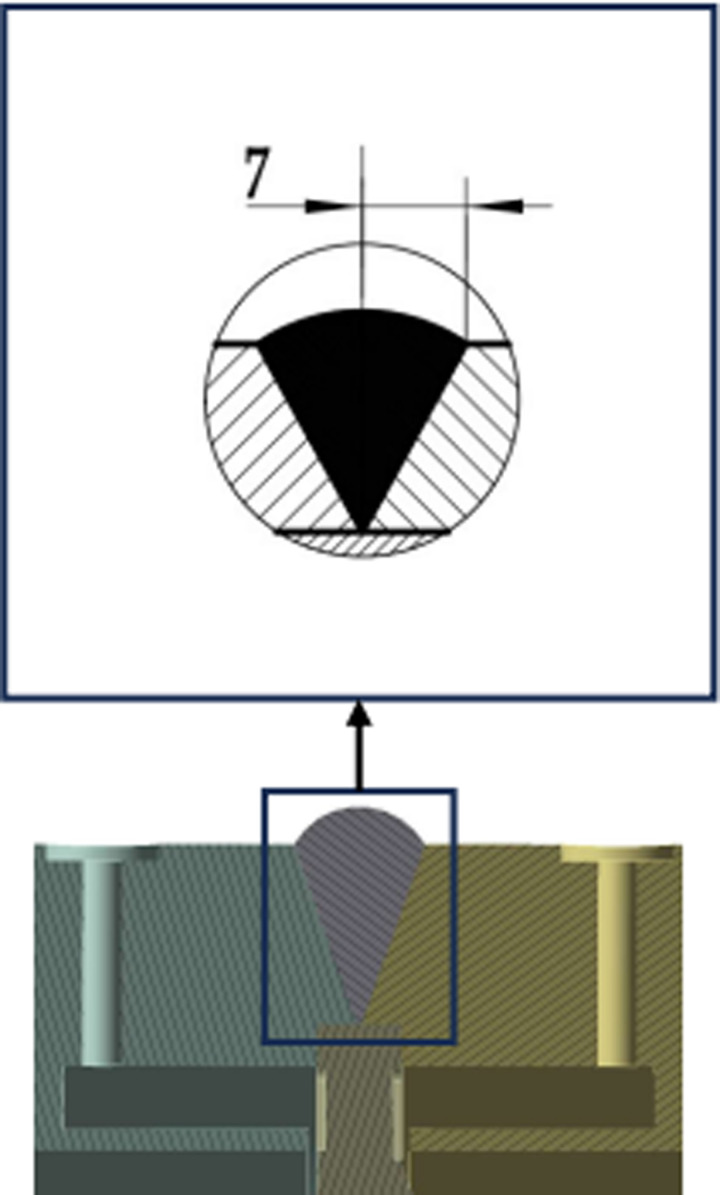
Section of Type Ⅳ weld structure.

The measuring medium is an air medium with a temperature of 700 °C, that is, the inner wall temperature is 700 °C; The flowmeter is in a normal temperature environment, with an outer wall temperature of 22 °C. There are four types of weld structures (I-IV) in the calculation model of this article [37], and each type of weld structure has three parameter combinations. The weld structure parameters are shown in Figs 3–6.

## 3. Results analysis

**Fig 7:**
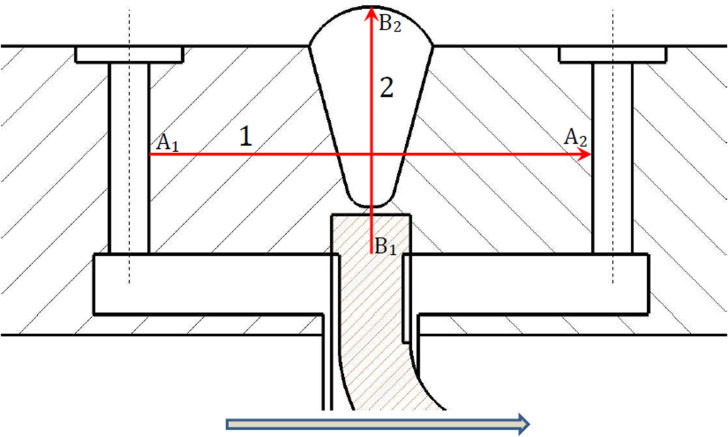
Schematic diagram of monitoring path.

A schematic of the temperature field monitoring path is shown in Fig 7 [37]. Monitoring path 1 is distributed along the axial direction, with the starting point A1 being the upstream pressure-taking port and the endpoint A2 being the downstream pressure-taking port. Monitoring path 2 is distributed perpendicular to the axis, with the starting point B1 near the inner wall and the endpoint B2 located on the outer wall. Points A1, A2, B1, and B2 are all midpoints of their respective structures.

### 3.1 Thermal stress characterization of monitoring path

**Fig 8:**
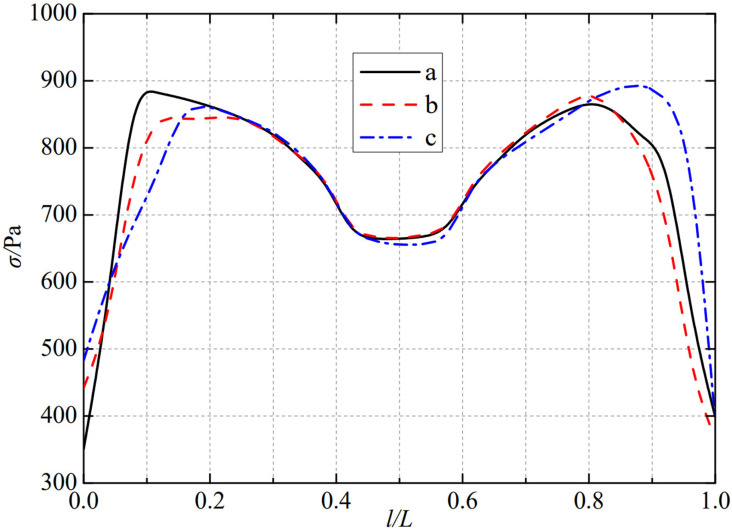
Thermal stress distribution of type I flow meter in monitoring path 1.

The thermal stress distribution in the monitoring path 1 of the Type I flowmeter is shown in Fig 8. In Type I-A, B, and C flow meters, the thermal stress distribution along the path from the upstream pressure-taking port to the downstream pressure-taking port shows a trend of first increasing, then decreasing, then increasing, and finally decreasing, and all reach the valley value of the gentle section of 0.4−0.6L at 0.5L, showing obvious symmetrical distribution properties. In monitoring path 1, the thermal stress of the three types of nozzle flow meters, a, b, and c, all showed a rapid increasing trend. Among them, the Type A nozzle flow meter first reached a peak of 882.33 Pa at 0.1L, the Type B nozzle flow meter reached a peak of 849.77 Pa at 0.1L and then entered a gentle range of 0.1−0.3L, and the Type C nozzle flow meter reached a peak of 843.72 Pa at 0.2L. In contrast, all three types of nozzle flowmeters, a, b, and c, show a rapid decrease in thermal stress after it peaks again. The thermal stress of Type A nozzle flowmeter reached its peak again at 0.8L, reaching 869.22 Pa. Type B nozzle flowmeter also reached its peak again at 0.8L, reaching 883.77 Pa. Type C flowmeter was slightly later than the first two types of flowmeter, reaching its peak again at 0.9L, reaching 895.86 Pa. The maximum valley values of monitoring path 1 for Type I-A, B, and C nozzle flow meters are 664.27 Pa, 663.94 Pa, and 654.66 Pa. From this, it can be seen that as the distance between the weld metal of the nozzle flowmeter and the eight slot nozzle increases, the thermal stress in its monitoring path 1 decreases in the valley value of the gentle section at 0.5L.

**Fig 9:**
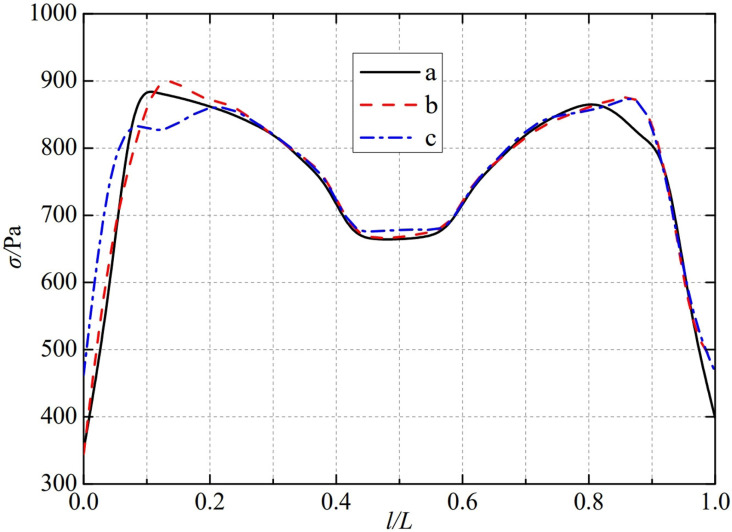
Thermal stress distribution of type II flow meter in monitoring path 1.

The thermal stress distribution in the monitoring path 1 of the Type II flowmeter is shown in Fig 9. In Type II-A, B, and C flow meters, the thermal stress distribution along the path from the upstream pressure-taking port to the downstream pressure-taking port shows a trend of first increasing, then decreasing, then increasing, and finally decreasing, and all reach the valley value of the gentle section of 0.4−0.6L at 0.5L, showing obvious symmetrical distribution properties. In monitoring path 1, the thermal stress of the three types of nozzle flow meters, a, b, and c, all showed a rapid increasing trend. Among them, the Type A nozzle flow meter first reached a peak of 882.33 Pa at 0.1L, the Type B nozzle flow meter reached a peak of 909.39 Pa at 0.12L, and the Type C nozzle flow meter reached a peak of 861.92 Pa at 0.23L. In contrast, all three types of nozzle flowmeters, a, b, and c, show a rapid decrease in thermal stress after it peaks again. The thermal stress of Type A nozzle flowmeter reached its peak again at 0.8L, reaching 869.22 Pa. Type B and Type C flowmeter was slightly later than the Type A flowmeter, Type B nozzle flowmeter reached its peak again at 0.88L, reaching 881.21 Pa, Type C nozzle flowmeter reached its peak again at 0.88L, reaching 884.14 Pa. The maximum valley values of monitoring path 1 for Type II-A, B, and C nozzle flow meters are 664.27 Pa, 666.78 Pa, and 678.2 Pa. From this, it can be seen that as the width below the weld metal of the nozzle flowmeter increases, the thermal stress in its monitoring path 1 also increases in the valley value of the gentle section at 0.5L.

**Fig 10:**
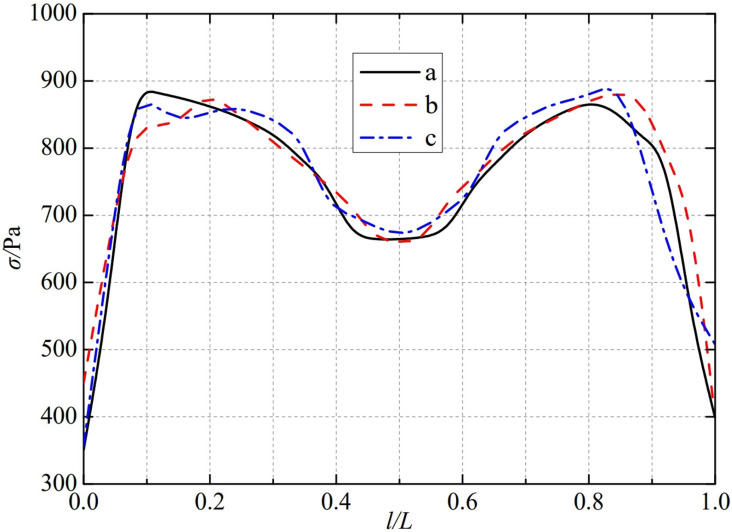
Thermal stress distribution of type Ⅲ flow meter in monitoring path 1.

The thermal stress distribution in the monitoring path 1 of the Type Ⅲ flowmeter is shown in Fig 10. In Type Ⅲ-A, B, and C flow meters, the thermal stress distribution along the path from the upstream pressure-taking port to the downstream pressure-taking port shows a trend of first increasing, then decreasing, then increasing, and finally decreasing, and all reach the valley value of the gentle section of 0.4−0.6L at 0.5L, showing obvious symmetrical distribution properties. In monitoring path 1, the thermal stress of the three types of nozzle flow meters, a, b, and c, all showed a rapid increasing trend. Among them, the Type A nozzle flow meter first reached a peak of 882.33 Pa at 0.1L, the Type B nozzle flow meter reached a peak of 882.13 Pa at 0.2L. The Type C nozzle flow meter reached a peak of 881.62 Pa at 0.08L, afterwards, there was a small fluctuation in the 0.1−0.3L range. In contrast, all three types of nozzle flowmeters, a, b, and c, show a rapid decrease in thermal stress after it peaks again. The thermal stress of Type A nozzle flowmeter reached its peak again at 0.8L, reaching 869.22 Pa. Type B and Type C flowmeter was slightly later than the Type A flowmeter, both peaked again at 0.85L for thermal stresses of 882.59 Pa and 898.71 Pa. The maximum valley values of monitoring path 1 for Type Ⅲ-A, B, and C nozzle flow meters are 664.27 Pa, 660.09 Pa, and 670 Pa. From this, it can be seen that as the conical angle of the weld metal in the nozzle flowmeter increases, the thermal stress in its monitoring path 1 also increases in the valley value of the gentle section at 0.5L.

**Fig 11:**
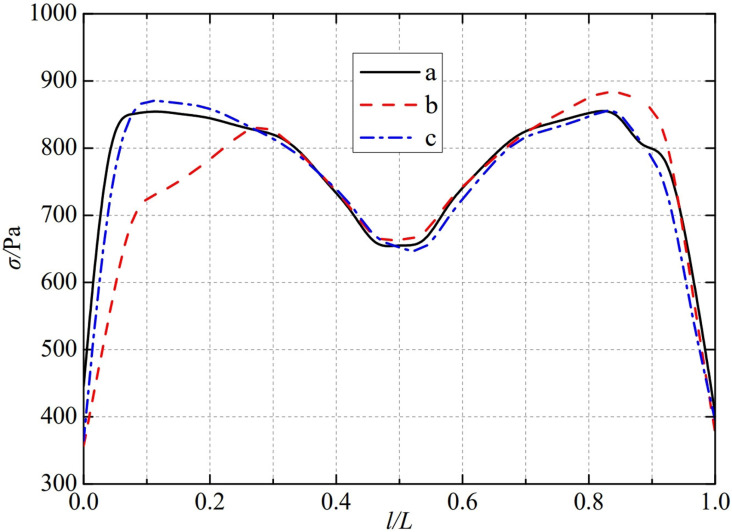
Thermal stress distribution of type Ⅳ flow meter in monitoring path 1.

The thermal stress distribution in the monitoring path 1 of the Type Ⅳ flowmeter is shown in Fig 11. In Type Ⅳ-A, B, and C flow meters, the thermal stress distribution along the path from the upstream pressure-taking port to the downstream pressure-taking port shows a trend of first increasing, then decreasing, then increasing, and finally decreasing, and all reach the valley value of the gentle section of 0.4−0.6L at 0.5L, showing obvious symmetrical distribution properties. In contrast, the width of the flat section of Type C flow meters is less than that of Type B flow meters and less than that of Type A flow meters. In monitoring path 1, the thermal stress of the three types of nozzle flow meters, a, b, and c, all showed a rapid increasing trend. Among them, Type IV-A and C nozzle flow meters both reached their peak at 0.11L, at 856.1 Pa and 870.97 Pa, while Type B nozzle flow meters experienced a rapid increase in thermal stress in the 0−0.1L range. After reaching 730.26 Pa at 0.1L, the rate of thermal stress increase slightly slowed down, and finally reached a peak of 835.54 Pa at 0.27L. Compared with Type A and C nozzle flow meters, the first peak position was delayed. In contrast, all three types of nozzle flowmeters, a, b, and c, show a rapid decrease in thermal stress after it peaks again. The thermal stress of the three types of nozzle flow meters, a, b and c, reached its peak again at 0.85L, reaching 858.36 Pa, 886.18 Pa, and 863.98 Pa. The maximum valley values of monitoring path 1 for Type Ⅳ-A, B, and C nozzle flow meters are 657.86 Pa, 664.93 Pa, and 639.12 Pa. From this, when the metal cone angle opening of the weld seam of the nozzle flowmeter is Type C, the thermal stress in its monitoring path 1 decreases significantly in the valley value of the gentle section at 0.5L.

**Fig 12:**
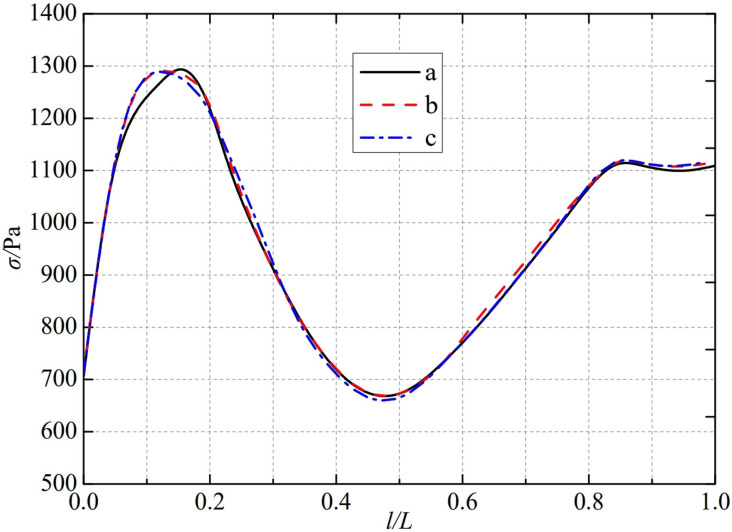
Thermal stress distribution of type I flow meter in monitoring path 2.

The thermal stress distribution in the monitoring path 2 of the Type I flowmeter is shown in Fig 12. In Type I-A, B, and C flow meters, the thermal stress distribution along the path from the inner wall to the outer wall shows a trend of first increasing, then decreasing, and finally increasing, and all reach the valley value at 0.46L. The thermal stress of Type A nozzle flowmeter reaches a peak of 1310.4 Pa at 0.15L, while Type B and Type C nozzle flowmeters are slightly earlier than Type A nozzle flowmeters. The thermal stress reaches a peak at 0.12L, at 1296.1 Pa and 1296.3 Pa. The maximum valley values of monitoring path 2 for Type I-A, B, and C nozzle flow meters are 666.04 Pa, 666.54 Pa, and 657.18 Pa, at the same time, the thermal stress changes tend to be gentle at 0.85L. From this, it can be seen that as the distance between the weld metal of the nozzle flowmeter and the eight slot nozzle increases, the minimum valley value of thermal stress in monitoring path 2 at Type C is relatively small.

**Fig 13:**
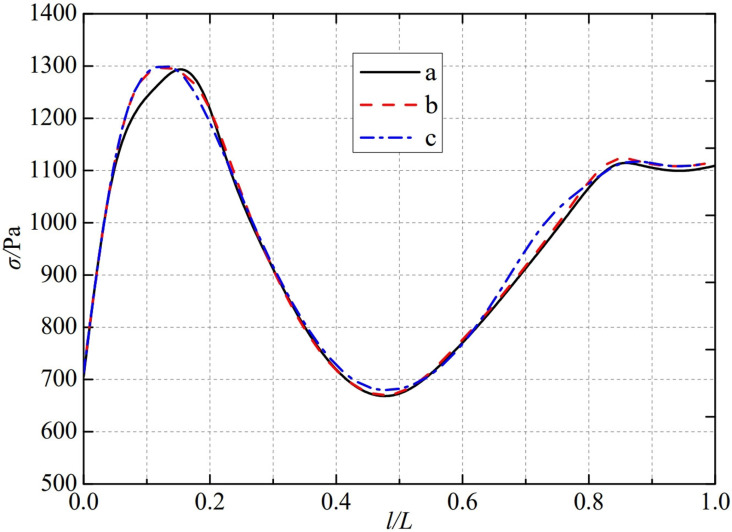
Thermal stress distribution of type II flow meter in monitoring path 2.

The thermal stress distribution in the monitoring path 2 of the Type II flowmeter is shown in Fig 13. In Type II-A, B, and C flow meters, the thermal stress distribution along the path from the inner wall to the outer wall shows a trend of first increasing, then decreasing, and finally increasing, and all reach the valley value at 0.46L. The thermal stress of Type A nozzle flowmeter reaches a peak of 1310.4 Pa at 0.15L, while Type B and Type C nozzle flowmeters are slightly earlier than Type A nozzle flowmeters. The thermal stress reaches a peak at 0.12L, at 1302.5 Pa and 1305.5 Pa. The maximum valley values of monitoring path 2 for Type II-A, B, and C nozzle flow meters are 666.04 Pa, 668.02 Pa, and 678.28 Pa, at the same time, the thermal stress changes tend to be gentle at 0.85L. From this, it can be seen that as the width below the weld metal of the nozzle flowmeter increases, the thermal stress in its monitoring path 2 also increases in the minimum valley value.

**Fig 14:**
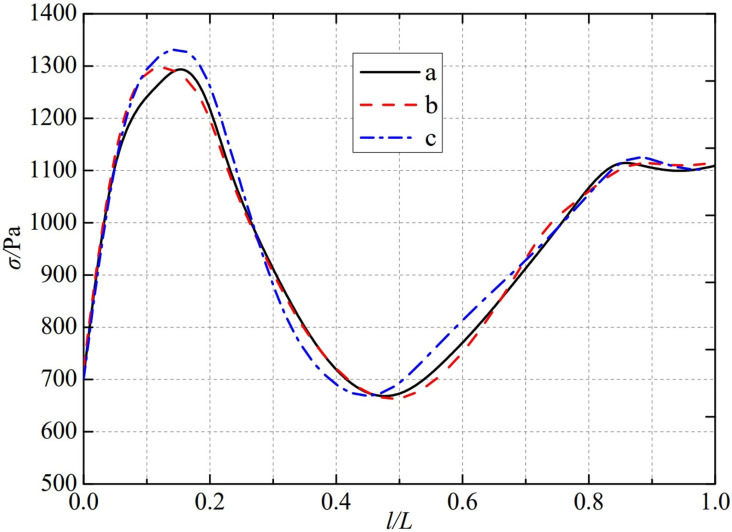
Thermal stress distribution of type Ⅲ flow meter in monitoring path 2.

The thermal stress distribution in the monitoring path 2 of the Type Ⅲ flowmeter is shown in Fig 14. In Type Ⅲ-A, B, and C flow meters, the thermal stress distribution along the path from the inner wall to the outer wall shows a trend of first increasing, then decreasing, and finally increasing, and all reach the valley value at 0.46L. The thermal stress of Type A and Type C nozzle flowmeter reaches a peak at 0.15L, at 1310.4 Pa and 1340.6 Pa. while Type B nozzle flowmeters are slightly earlier than Type A and Type C nozzle flowmeters. The thermal stress reaches a peak at 0.12L, at 1302.9 Pa. The maximum valley values of monitoring path 2 for Type Ⅲ-A, B, and C nozzle flow meters are 666.04 Pa, 660.4 Pa, and 666.13 Pa, at the same time, the thermal stress changes tend to be gentle at 0.85L. From this, it can be seen that as the conical angle of the weld metal in the nozzle flowmeter increases, the minimum valley value of thermal stress in monitoring path 2 at Type B is relatively small.

**Fig 15:**
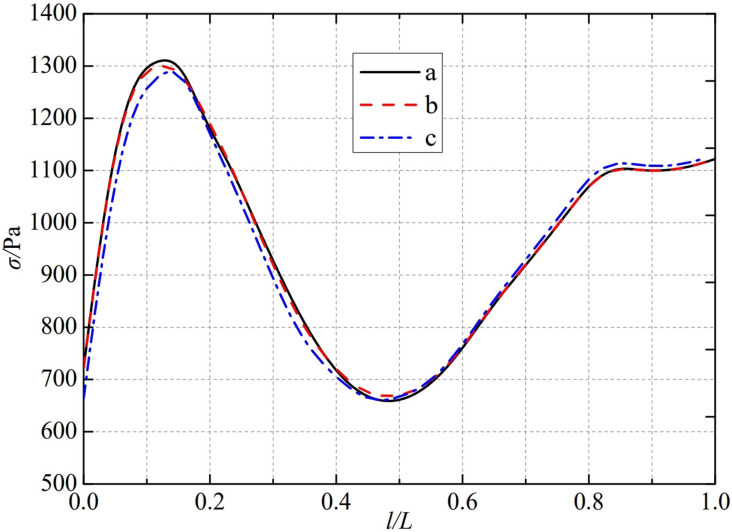
Thermal stress distribution of type Ⅳ flow meter in monitoring path 2.

The thermal stress distribution in the monitoring path 2 of the Type Ⅳ flowmeter is shown in Fig 15. In Type Ⅳ-A, B, and C flow meters, the thermal stress distribution along the path from the inner wall to the outer wall shows a trend of first increasing, then decreasing, and finally increasing, and all reach the valley value at 0.46L. The thermal stress of Type A and Type B nozzle flowmeter reaches a peak at 0.12L, at 1314.1 Pa and 1305.5 Pa. while Type C nozzle flowmeters are slightly earlier than Type A and Type B nozzle flowmeters. The thermal stress reaches a peak at 0.15L, at 1300.8 Pa. The maximum valley values of monitoring path 2 for Type Ⅳ-A, B, and C nozzle flow meters are 657.35 Pa, 667.33 Pa, and 656.25 Pa, at the same time, the thermal stress changes tend to be gentle at 0.85L. From this, when the metal cone angle opening of the weld seam of the nozzle flowmeter is Type B, the minimum valley value of thermal stress is relatively small.

### 3.2 Thermal stress in solid domain

**Fig 16:**
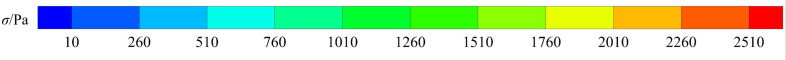
Section thermal stress in Type I flow meter (Pa). (a) Type I-A weld structure (b) Type I-B weld structure (c) Type I-C weld structure.

Fig 16 shows the distribution of thermal stresses in the cross section in the Type I flowmeter. When the temperature of the outer wall remains constant at 20 °C and the temperature of the inner wall rises to 700 °C, the internal thermal stress values of Type I-A, B, and C nozzle flow meters all show significant increases. The phenomenon of stress concentration is mainly concentrated near the upstream and downstream pressure-taking ports and the inlet and outlet of the flow meter, with a maximum stress value of 2510 Pa. And the thermal stress distribution inside Type I-A, B, and C flow meters is similar, which indicates that the distance between the weld metal and the eight groove nozzle has less influence on the overall thermal stress and stress concentration phenomenon inside the flowmeter.

**Fig 17:**
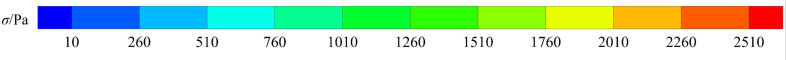
Section thermal stress in Type II flow meter (Pa). (a) Type II-A weld structure (b) Type II-B weld structure (c) Type II-C weld structure.

Fig 17 shows the distribution of thermal stresses in the cross section in the Type II flowmeter. When the temperature of the outer wall remains constant at 20 °C and the temperature of the inner wall rises to 700 °C, the internal thermal stress values of Type II-A, B, and C nozzle flow meters all show significant increases. The phenomenon of stress concentration is mainly concentrated near the upstream and downstream pressure-taking ports and the inlet and outlet of the flow meter, with a maximum stress value of 2510 Pa. And the thermal stress distribution inside Type II-A, B, and C flow meters is similar, which indicates that the width below the weld metal of the nozzle flowmeter has less influence on the overall thermal stress and stress concentration phenomenon inside the flowmeter.

**Fig 18:**
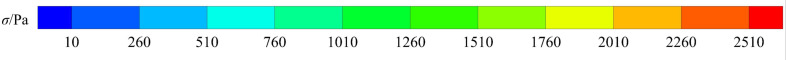
Section thermal stress in Type Ⅲ flow meter (Pa). (a) Type Ⅲ-A weld structure (b) Type Ⅲ-B weld structure (c) Type Ⅲ-C weld structure.

Fig 18 shows the distribution of thermal stresses in the cross section in the Type Ⅲ flowmeter. When the temperature of the outer wall remains constant at 20 °C and the temperature of the inner wall rises to 700 °C, the internal thermal stress values of Type Ⅲ-A, B, and C nozzle flow meters all show significant increases. The phenomenon of stress concentration is mainly concentrated near the upstream and downstream pressure-taking ports and the inlet and outlet of the flow meter, with a maximum stress value of 2510 Pa. The thermal stress distribution at the inlet and outlet of Type Ⅲ-C flow meters is slightly smaller than that of Type A and B flow meters, indicating that the conical angle of the weld metal has a certain influence on the overall thermal stress and stress concentration phenomenon inside the flow meter. As the conical angle of the weld metal increases, the stress value at the inlet and outlet of the flow meter decreases.

**Fig 19:**
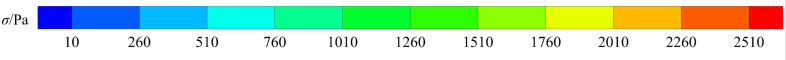
Section thermal stress in Type Ⅳ flow meter (Pa). (a) Type Ⅳ-A weld structure (b) Type Ⅳ-B weld structure (c) Type Ⅳ-C weld structure.

Fig 19 shows the distribution of thermal stresses in the cross section in the Type Ⅳ flowmeter. When the temperature of the outer wall remains constant at 20 °C and the temperature of the inner wall rises to 700 °C, the internal thermal stress values of Type Ⅳ-A, B, and C nozzle flow meters all show significant increases. The phenomenon of stress concentration is mainly concentrated near the upstream and downstream pressure-taking ports and the inlet and outlet of the flow meter, with a maximum stress value of 2510 Pa. And the thermal stress distribution inside Type Ⅳ-A, B, and C flow meters is similar, which indicates that the opening of the weld metal cone angle has less influence on the overall thermal stress and stress concentration phenomenon inside the flowmeter.

### 3.3 Thermal deformation of monitoring path

**Fig 20:**
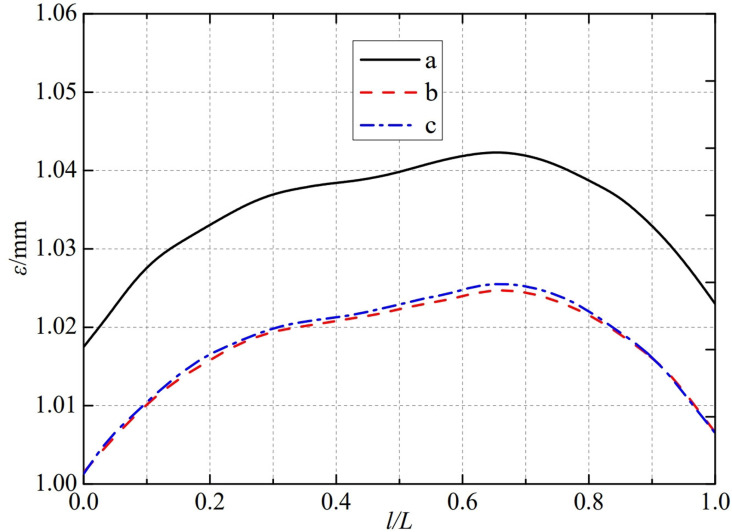
Thermal deformation distribution of type I flow meter in monitoring path 1.

The thermal deformation distribution in the monitoring path 1 of the Type I flowmeter is shown in Fig 20. In Type I-A, B, and C flow meters, the thermal deformation distribution along the path from the upstream pressure-taking port to the downstream pressure-taking port shows a trend of first increasing, then decreasing, and all reach the maximum peak value at 0.65L. The maximum peak values of monitoring path 1 for Type I-A, B, and C nozzle flow meters are 1.0424 mm, 1.0248 mm, and 1.0256 mm. In this case, the difference in peak thermal deformation between flowmeter Type A and Type B is only 0.0176 mm, and the difference in peak thermal deformation between flowmeter Type B and Type C is only 0.0008 mm. From this, it can be seen that as the distance between the weld metal of the nozzle flowmeter and the eight-slot nozzle increases, the difference between the thermal deformation curves is gradually negligible. The distance between the weld metal of the nozzle flowmeter and the eight-slot nozzle has a relatively small impact on the thermal deformation peak of monitoring path 1.

**Fig 21:**
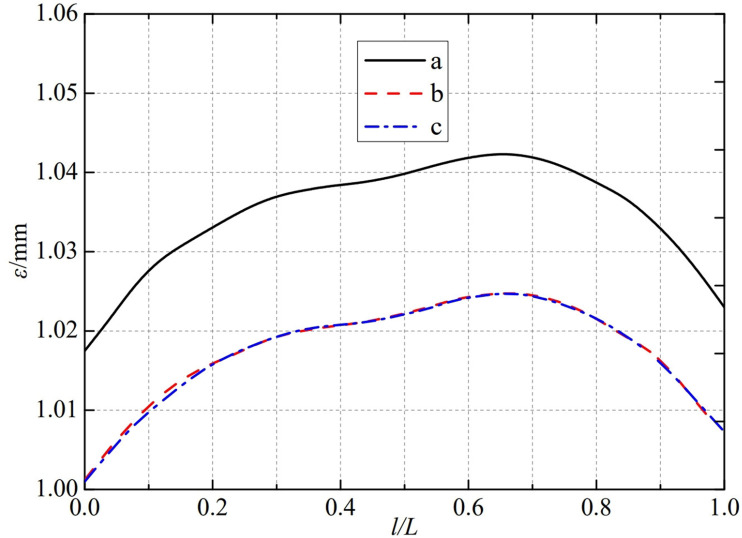
Thermal deformation distribution of type II flow meter in monitoring path 1.

The thermal deformation distribution in the monitoring path 1 of the Type II flowmeter is shown in Fig 21. In Type II-A, B, and C flow meters, the thermal deformation distribution along the path from the upstream pressure-taking port to the downstream pressure-taking port shows a trend of first increasing, then decreasing, and all reach the maximum peak value at 0.65L. The maximum peak values of monitoring path 1 for Type II-A, B, and C nozzle flow meters are 1.0424 mm, 1.0248 mm, and 1.0248 mm. In this case, the difference in peak thermal deformation between flowmeter Type A and Type B is only 0.0176 mm, and the peak value of thermal deformation has almost no difference between flowmeter Type B and Type C. From this, it can be seen that as the width below the weld metal of the nozzle flowmeter increases, the difference between the thermal deformation curves is gradually negligible. The width below the weld metal of the nozzle flowmeter has a relatively small impact on the thermal deformation peak of monitoring path 1.

**Fig 22:**
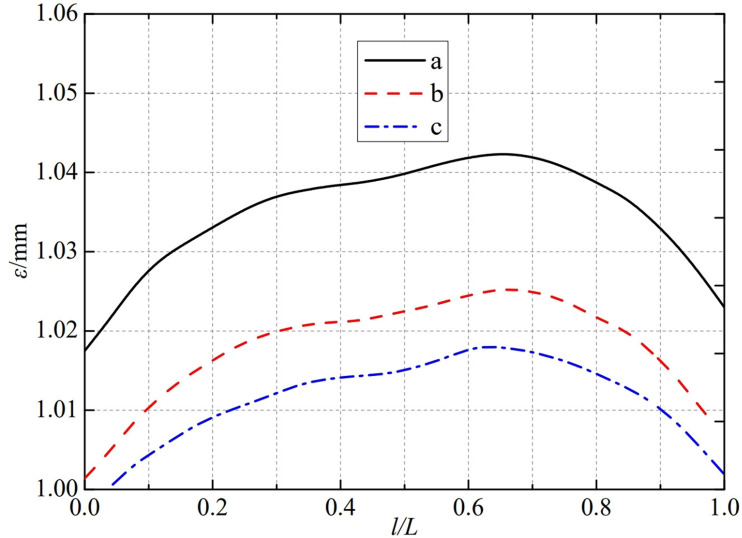
Thermal deformation distribution of type Ⅲ flow meter in monitoring path 1.

The thermal deformation distribution in the monitoring path 1 of the Type Ⅲ flowmeter is shown in Fig 22. In Type Ⅲ-A, B, and C flow meters, the thermal deformation distribution along the path from the upstream pressure-taking port to the downstream pressure-taking port shows a trend of first increasing, then decreasing, and all reach the maximum peak value at 0.65L. The maximum peak values of monitoring path 1 for Type Ⅲ-A, B, and C nozzle flow meters are 1.0424 mm, 1.0253 mm, and 1.0181 mm. In this case, the difference in peak thermal deformation between flowmeter Type A and Type B is only 0.0171 mm, and the difference in peak thermal deformation between flowmeter Type B and Type C is only 0.0072 mm. From this, it can be seen that as the conical angle of the weld metal in the nozzle flowmeter increases, the peak thermal deformation of monitoring path 1 decreases accordingly.

**Fig 23:**
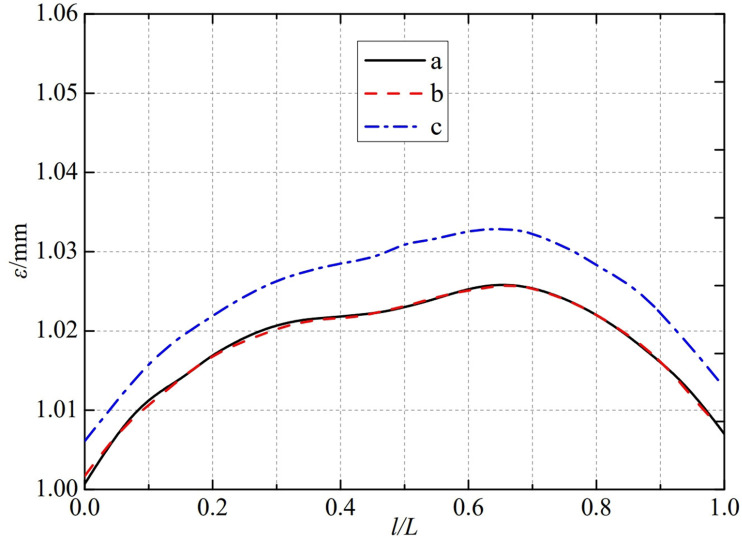
Thermal deformation distribution of type Ⅳ flow meter in monitoring path 1.

The thermal deformation distribution in the monitoring path 1 of the Type Ⅳ flowmeter is shown in Fig 23. In Type Ⅳ-A, B, and C flow meters, the thermal deformation distribution along the path from the upstream pressure-taking port to the downstream pressure-taking port shows a trend of first increasing, then decreasing, and all reach the maximum peak value at 0.65L. The maximum peak values of monitoring path 1 for Type Ⅳ-A, B, and C nozzle flow meters are 1.0259 mm, 1.0258 mm, and 1.0329 mm. In this case, the difference in peak thermal deformation between flowmeter Type A and Type B is only 0.0001 mm, and the difference in peak thermal deformation between flowmeter Type B and Type C is only 0.0071 mm. From this, the metal cone angle opening of the weld seam of the nozzle flowmeter has a relatively small impact on the thermal deformation peak of monitoring path 1.

**Fig 24:**
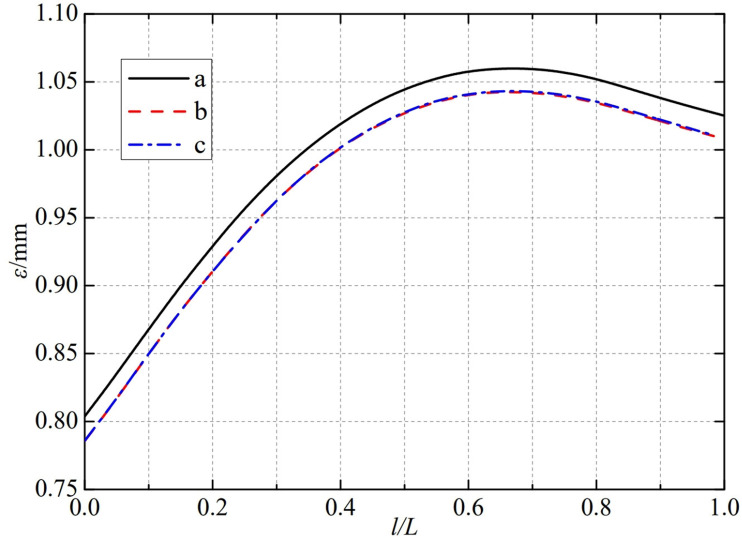
Thermal deformation distribution of type I flow meter in monitoring path 2.

The thermal deformation distribution in the monitoring path 2 of the Type I flowmeter is shown in Fig 24. In Type I-A, B, and C flow meters, the thermal deformation distribution along the path from the inner wall to the outer wall shows a trend of first increasing, then decreasing, and all reach the maximum peak value at 0.65L. The maximum peak values of monitoring path 2 for Type I-A, B, and C nozzle flow meters are 1.0599 mm, 1.0426 mm, and 1.0434 mm. In this case, the difference in peak thermal deformation between flowmeter Type A and Type B is only 0.0173 mm, and the difference in peak thermal deformation between flowmeter Type B and Type C is only 0.0008 mm. From this, the distance between the weld metal of the nozzle flowmeter and the eight-slot nozzle has a relatively small impact on the thermal deformation peak of monitoring path 2.

**Fig 25:**
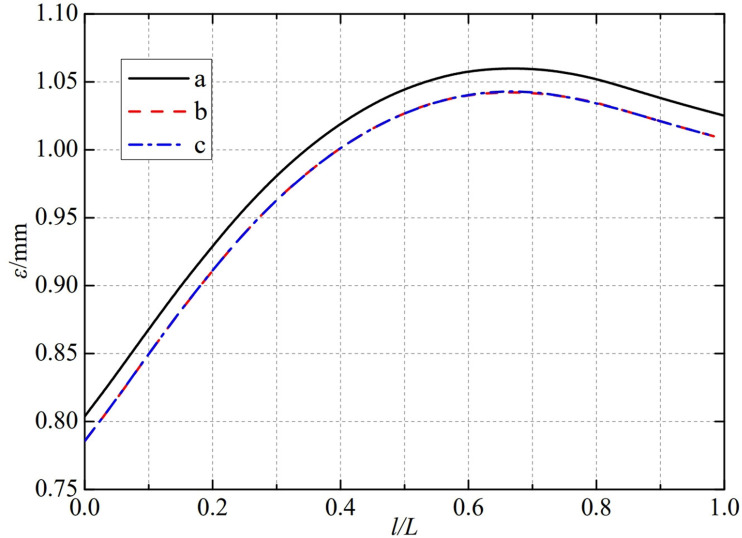
Thermal deformation distribution of type II flow meter in monitoring path 2.

The thermal deformation distribution in the monitoring path 2 of the Type II flowmeter is shown in Fig 25. In Type II-A, B, and C flow meters, the thermal deformation distribution along the path from the inner wall to the outer wall shows a trend of first increasing, then decreasing, and all reach the maximum peak value at 0.65L. The maximum peak values of monitoring path 2 for Type II-A, B, and C nozzle flow meters are 1.0599 mm, 1.0423 mm, and 1.0431 mm. In this case, the difference in peak thermal deformation between flowmeter Type A and Type B is only 0.0176 mm, and the difference in peak thermal deformation between flowmeter Type B and Type C is only 0.0008 mm. From this, the width below the weld metal of the nozzle flowmeter has a relatively small impact on the thermal deformation peak of monitoring path 2.

**Fig 26:**
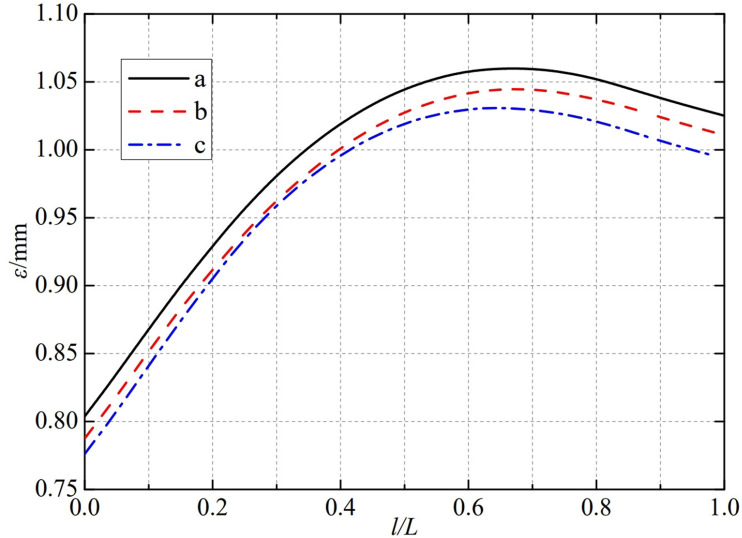
Thermal deformation distribution of type Ⅲ flow meter in monitoring path 2.

The thermal deformation distribution in the monitoring path 2 of the Type Ⅲ flowmeter is shown in Fig 26. In Type Ⅲ-A, B, and C flow meters, the thermal deformation distribution along the path from the inner wall to the outer wall shows a trend of first increasing, then decreasing, and all reach the maximum peak value at 0.65L. The maximum peak values of monitoring path 2 for Type Ⅲ-A, B, and C nozzle flow meters are 1.0599 mm, 1.0447 mm, and 1.0308 mm. In this case, the difference in peak thermal deformation between flowmeter Type A and Type B is only 0.0152 mm, and the difference in peak thermal deformation between flowmeter Type B and Type C is only 0.0139 mm. From this, as the conical angle of the weld metal in the nozzle flowmeter increases, the peak thermal deformation of monitoring path 2 decreases, but the overall impact is relatively small.

**Fig 27:**
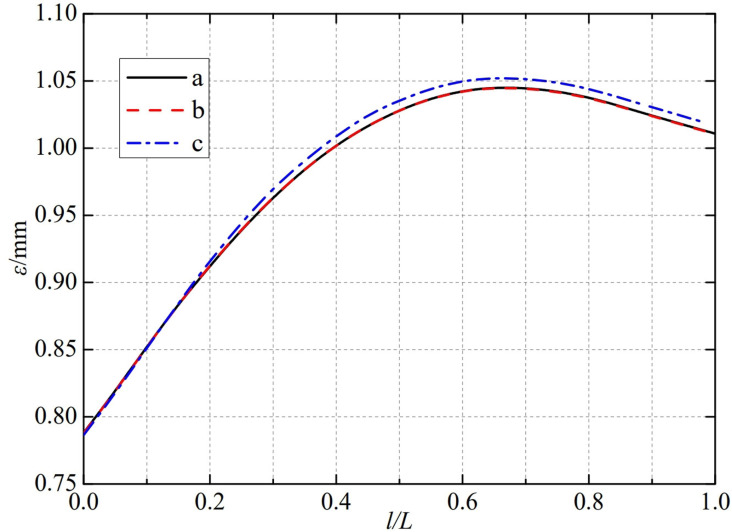
Thermal deformation distribution of type Ⅳ flow meter in monitoring path 2.

The thermal deformation distribution in the monitoring path 2 of the Type Ⅳ flowmeter is shown in Fig 27. In Type Ⅳ-A, B, and C flow meters, the thermal deformation distribution along the path from the inner wall to the outer wall shows a trend of first increasing, then decreasing, and all reach the maximum peak value at 0.65L. The maximum peak values of monitoring path 2 for Type Ⅳ-A, B, and C nozzle flow meters are 1.0451 mm, 1.0448 mm, and 1.0522 mm. In this case, the difference in peak thermal deformation between flowmeter Type A and Type B is only 0.0003 mm, and the difference in peak thermal deformation between flowmeter Type B and Type C is only 0.0074 mm. From this, the metal cone angle opening of the weld seam of the nozzle flowmeter has a relatively small impact on the thermal deformation peak of monitoring path 2.

### 3.4 Thermal deformation in the solid domain

**Fig 28:**
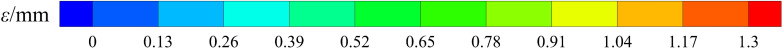
Section thermal deformation in Type I flow meter (mm). (a) Type I-A weld structure (b) Type I-B weld structure (c) Type I-C weld structure.

Fig 28 shows the distribution of thermal deformation in the cross section in the Type I flowmeter. When the temperature of the outer wall remains constant at 20 °C and the temperature of the inner wall rises to 700 °C, the thermal deformation values in the larger thermal deformation areas of the upstream and downstream pressure-taking ports and the inlet and outlet of the eight slot nozzle of Type I-A, B, and C nozzle flow meters all showed significant increases, with a maximum thermal deformation value of 1.3 mm. And the thermal deformation distribution inside Type I-A, B, and C flow meters is similar, which indicates that the distance between the weld metal and the eight-groove nozzle has less influence on the overall thermal deformation inside the flowmeter.

**Fig 29:**
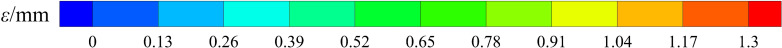
Section thermal deformation in Type II flow meter (mm). (a) Type II-A weld structure (b) Type II-B weld structure (c) Type II-C weld structure.

Fig 29 shows the distribution of thermal deformation in the cross section in the Type II flowmeter. When the temperature of the outer wall remains constant at 20 °C and the temperature of the inner wall rises to 700 °C, the thermal deformation values in the larger thermal deformation areas of the upstream and downstream pressure-taking ports and the inlet and outlet of the eight slot nozzle of Type II-A, B, and C nozzle flow meters all showed significant increases, with a maximum thermal deformation value of 1.3 mm. And the thermal deformation distribution inside Type II-A, B, and C flow meters is similar, which indicates that the width below the weld metal of the nozzle flowmeter has less influence on the overall thermal deformation inside the flowmeter.

**Fig 30:**
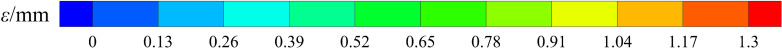
Section thermal deformation in Type Ⅲ flow meter (mm). (a) Type Ⅲ-A weld structure (b) Type Ⅲ-B weld structure (c) Type Ⅲ-C weld structure.

Fig 30 shows the distribution of thermal deformation in the cross section in the Type Ⅲ flowmeter. When the temperature of the outer wall remains constant at 20 °C and the temperature of the inner wall rises to 700 °C, the thermal deformation values in the larger thermal deformation areas of the upstream and downstream pressure-taking ports and the inlet and outlet of the eight slot nozzle of Type Ⅲ-A, B, and C nozzle flow meters all showed significant increases, with a maximum thermal deformation value of 1.3 mm. And the thermal deformation distribution inside Type Ⅲ-A, B, and C flow meters is similar, which indicates that the conical angle of the weld metal has less influence on the overall thermal deformation inside the flowmeter.

**Fig 31:**
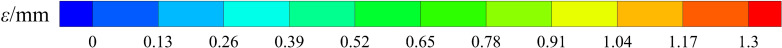
Section thermal deformation in Type Ⅳ flow meter (mm). (a) Type Ⅳ-A weld structure (b) Type Ⅳ-B weld structure (c) Type Ⅳ-C weld structure.

Fig 31 shows the distribution of thermal deformation in the cross section in the Type Ⅳ flowmeter. When the temperature of the outer wall remains constant at 20 °C and the temperature of the inner wall rises to 700 °C, the thermal deformation values in the larger thermal deformation areas of the upstream and downstream pressure-taking ports and the inlet and outlet of the eight slot nozzle of Type Ⅳ-A, B, and C nozzle flow meters all showed significant increases, with a maximum thermal deformation value of 1.3 mm. And the thermal deformation distribution inside Type Ⅳ-A, B, and C flow meters is similar, which indicates that the opening of the weld metal cone angle has less influence on the overall thermal deformation inside the flowmeter.

## 4. Conclusion

(1) Along the direction of medium flow, thermal stress shows a trend of first rapidly increasing, then slowly decreasing, then slowly increasing, and finally rapidly decreasing; The thermal deformation shows a slow increase followed by a slow decrease trend.
(2) In the direction from the inside to the outside, the thermal stresses all show a tendency to increase rapidly first, then decrease rapidly, and then increase rapidly again; the thermal deformation shows a tendency to increase rapidly first and then decrease gradually.
(3) The distance between the weld metal and the eight-groove nozzle (Type I), the size of the width of the bottom of the weld metal (Type II), and the variation of the taper angle opening of the weld metal (Type IV) all have a small effect on the overall thermal stress and thermal deformation of the nozzle flowmeter.
(4) As the conical angle of the weld metal (Type III) increases, the thermal stress at the inlet and outlet of the nozzle flowmeter gradually decreases, while the overall thermal deformation of the nozzle flowmeter changes less.
